# On-top arginine supplementation during lactation affects milk composition, performance, and intestinal bacterial and viral microbial community of sows and their piglets

**DOI:** 10.1093/jas/skaf319

**Published:** 2025-09-16

**Authors:** Luise Diana, Colitti Barbara, Correa Federico, Simongiovanni Aude, Bertolotti Luigi, Trevisi Paolo

**Affiliations:** Department of Agricultural and Food Sciences, University of Bologna, Bologna 40127, Italy; Department of Veterinary Sciences, University of Turin, Turin 10124, Italy; Department of Agricultural and Food Sciences, University of Bologna, Bologna 40127, Italy; Eurolysine Avril, Paris 75017, France; Department of Veterinary Sciences, University of Turin, Turin 10124, Italy; Department of Agricultural and Food Sciences, University of Bologna, Bologna 40127, Italy

**Keywords:** *Collinsella*, Cremvirales, functional amino acids, lactose, microbiota, mortality

## Abstract

Early gut microbiome colonization is crucial for gut physiology, immunity, and growth. It is influenced by factors like the maternal microbiome, which can be modified through diet, including amino acids (AAs) supplementation. Among AAs, arginine (Arg) is known to play a key role in lactating sows, which has attracted increased interest recently. The aim of this study was to investigate whether dietary supplementation of lactating sows with on-top Arg (22 g/d/sow) could influence the productive performance and milk of sows and their litters and their gut microbial community, including both viruses and bacteria. Thirty-two sows were divided into two groups balanced for parity and body weight: 1) control (CO) (fed a corn-based diet) and 2) CO + 22.5 g/d/sow of on-top Arg (ARG). Diets were fed from 4 d before farrowing (d4) to weaning (d27). Piglets were weighed at d0, d7, d14, d27, d34, and d41. Colostrum and milk were sampled at farrowing, d10, and d20 from proximal and immunoglobulin composition. Fecal and cecal samples were collected at d27 from sows and piglets (8 piglets/group), respectively. Arg increased the lactose content of milk collected at d20 (*P* = 0.05), favored the average daily gain of piglets from d0 to d41 (*P* = 0.04), and tended to reduce their mortality (*P* = 0.08). The gut microbiome of sows and piglets differed significantly in terms of bacterial and viral communities (beta diversity, *P* < 0.05). Bacteriophage composition differed markedly between sows and piglets, with higher Petitvirales in sows and Caudovirales in piglets (*P* < 0.01). Caudovirales positively correlated with *Subdoligranulum*, *Ruminococcus*, *Romboutsia*, and *Marvinbryantia* (r > 0.5; *P* < 0.05), which were also more abundant in piglets (*P* < 0.05). Arg did not affect the fecal microbial community of sows, whereas it increased the alpha diversity of the cecal bacterial (Shannon, *P* = 0.08) and viral (Shannon, *P* < 0.01) community of piglets. Piglets from ARG dams had a higher abundance of *Collinsella* (linear discriminant analysis [LDA] score = 4.16, *P*.adj = 0.05) and Cremvirales (*P* = 0.09) and an absence of Piccovirales (*P* = 0.07). In conclusion, the present study confirms the interest of Arg for lactating sows to promote piglet growth and intestinal eubiosis. The study shows that Arg administration can indirectly modify the microbiota profile of piglets at weaning. Finally, the results obtained between the viral and bacterial communities highlight the strong interplay between them, likely mediated by bacteriophages, warranting further investigation.

## Teaser

Supplementing lactating sows with arginine improves piglet growth, reduces mortality, and modifies their gut microbiome, highlighting the important role of maternal nutrition in shaping neonatal health.

Viral and bacterial communities highlight the strong interplay that appears to be controlled by bacteriophages and varies according to productive phases.

## Introduction

Early life microbial colonization of the gut plays a key role in mammalian health and performance (Madan et al., 2012; Korpela and de Vos, 2018). Correct early colonization of the gut is particularly important in mammals that are born immunodeficient, including piglets (Schokker et al., 2014; Mach et al., 2015). In fact, in pigs, maternal immune cells and immunoglobulins cannot pass from the mother to the fetus during pregnancy because the placenta of sows is epitheliochorial; therefore, neonatal piglets are considered immunodeficient at birth (Rooke and Bland, 2002). As in other mammals, the colonization of the pig intestine at birth starts with the first contact with the microbes in the sow’s vaginal tract and subsequently with the microbes in the sow’s feces and the environment (Hou et al., 2015). Recently, it has also been suggested that initial bacterial transfer from sows to piglets may occur via the umbilical cord (Leblois et al., 2018), but data are inconsistent (Palumbo et al., 2023). According to what is observed in other species such as humans, an additional pathway for newborn gut imprinting is through the vertical mother–newborn transmission by breastfeeding, and more recently, the entero–mammary pathway theory, also known as the gut–breast axis, has been considered (Vélez-Ixta et al., 2024). However, to the best of our knowledge, this pathway has not yet been fully confirmed in swine, as only a few studies have dealt with analyses of the microbiota of porcine colostrum and milk and its association with the gut microbiota of the piglets (Luise et al., 2025).

After the initial colonization, the composition of the porcine gut microbiome, which includes bacteria, eukaryotic viruses, bacterial viruses (bacteriophages), fungi, and archaea, changes during the next maturation steps towards a stereotypical “adult-like” microbial community structure (Bian et al., 2016; Trevisi et al., 2021). This process can be influenced by multiple interacting factors, including diet, animal genetics, immune maturation of each specific animal, management and environmental factors, and antibiotic use (Trevisi et al., 2021).

Manipulation of the gut microbiome through feeding and nutritional strategies has been widely considered a valid and rapid strategy for livestock. Promising effects on gut microbial entablement can be achieved using targeted feeding and nutritional strategies applied to piglets at birth and during lactation (Luise et al., 2021b). Other studies have suggested that nutritional and feeding strategies, including the use of resistant starch, prebiotics, and probiotics, can be applied to sow diets, resulting in a significant effect on the microbial profile as well as the growth and survivability of their offspring in the short and long term (Baker et al., 2013; Hasan et al., 2018; Leblois et al., 2018). More recently, amino acids **(AAs)** have been suggested to play an important role as microbial modulators (Spring et al., 2020; Chalvon-Demersay et al., 2021; Luise et al., 2022). Furthermore, according to Beaumont et al. (2022), AAs can also participate in the production of bioactive metabolites and positively influence host health, so the authors introduced the concept of “aminobiotics,” which refers to the functional role of some AAs to act as prebiotics. Among AAs, arginine **(Arg)** plays a crucial role in lactating sows; it can stimulate the secretion of key anabolic hormones, including insulin and growth hormone, and reduce the catabolic status of sows during the lactation period (Kim et al., 2004; Mateo et al., 2008), and also promote the production of prolactin (Kim et al., 2004; Mateo et al., 2008), which can stimulate the development of the mammary gland and the milk synthesis (Chew et al., 1984; Krogh et al., 2016). In addition, Arg can be metabolized to nitric oxide, which can increase blood flow and milk production by the mammary glands of lactating sows and promote the production of amines and polyamines in milk, which in turn can promote the mucosal development of piglets (Wu et al., 2009). Our previous study showed that supplementation of lactating sows with top-quality Arg (22 g/d/sow) could increase prolactin and immunoglobulin M in sow blood, monocyte percentage in piglet blood during suckling, and reduce piglet intestinal inflammation, resulting in a reduction in piglet mortality (Luise et al., 2023). Furthermore, studies suggested that Arg can modulate the fecal bacterial community of pregnant and lactating sows (Luise et al., 2020; Luise et al., 2023). However, much less is known about the effect of AAs, and in particular Arg, on the viral microbiome (virome), a diverse community consisting of eukaryotic RNA and DNA viruses and bacteriophages. Nevertheless, emerging evidence suggests that the virome plays a role in health and has important cross-talk with bacteria and the host, as recently demonstrated in humans (Iorio et al., 2022), and that the viral community can be modulated by diet (Howe et al., 2016).

Therefore, in the present study, it was hypothesized that supplementation of Arg in the diet of lactating sows may modulate not only the bacterial but also the viral microbial community of the sow and that this modulatory effect on the microbiota may also contribute to modifying the metabolism of the sow by changing the composition of colostrum and milk. Subsequently, these changes in the gut microbial profile and milk may lead to improvements in microbiota establishment, health, and growth of piglets suckled by arginine-supplemented sows.

This study aims to investigate the effect of dietary supplementation of Arg above the dietary requirement on the performance, colostrum and milk composition, and gut microbiome, including both bacterial and viral communities of lactating sows and their litters.

## Material and Methods

### Ethics

The animals included in the present study were sows and piglets reared under conventional conditions in the European Union **(EU)** according to Dir. 120/2008 EC. The Italian Ministry of Health approved the experimental procedures of the study with protocol No. 517/2018-PR.

### Experimental design and sampling

Sows and piglets included in the trial were housed in conventional farrowing crates with a nest area consisting of the entire floor under a warm lamp. Animals were recruited from three consecutive batches. Four days before farrowing (d-4), a total of 32 sows were divided into two groups (16 sows per group) according to parity (gilts were not included) and body weight **(BW)**: 1) a control **(CO)** group fed a standard lactating sow diet and 2) an Arg **(ARG)** group fed a standard lactating sow diet plus 22.5 g/d/sow. The Arg supplementation was added to the diet once daily during the morning meal. The standard diet for lactating sows and its calculated and analyzed composition are reported in Table 1.

**Table 1.** Composition, nutrient components, and analyzed values of the diet for lactating sows

Ingredients, %	
Corn	27.5
Barley	22.0
Soft wheat bran	21.0
Soybean meal 50% CP	13.5
Soft wheat	6.0
Rice hulls	3.3
Toasted full-fat soybeans	2.0
Pressed beet pulp	1.5
Calcium carbonate	1.2
Dicalcium phosphate (hydrated)	0.9
Premix^1^	0.5
Sodium chloride	0.3
L-Lysine HCl	0.3
DL-Methionine	0.1
Calculated composition	
Dry matter, %	85.81
Ash, %	5.48
Crude protein, %	16.02
Crude fat, %	3.37
Crude fiber, %	4.62
Neutral detergent fiber, %	18.33
Acid detergent fiber, %	5.76
Starch, %	37.80
Sugars, %	3.99
Metabolizable energy, MJ/kg	12.35
Calcium, %	0.78
Phosphorus, %	0.68
Analyzed composition, % feed	
Dry matter	89.24
Crude protein	16.20
Lysine	0.87
Threonine	0.57
Methionine	0.32
Cysteine	0.28
Methionine + cysteine	0.60
Tryptophan	0.20
Valine	0.74
Isoleucine	0.60
Leucine	1.18
Arginine	0.97
Phenylalanine	0.73
Tyrosine	0.51
Histidine	0.40
Serine	0.75
Alanine	0.76
Aspartic acid	1.34
Glutamic acid	3.08
Glycine	0.70
Proline	1.07

1 Premix provided the following per kilogram of premix: Vitamins: A, 10,000 IU; D3, 1,500 IU; E, 110 mg; B1, 312 mg; B5, 10 mg; B12, 0.015 mg; biotin, 0.1 mg; niacin, 20 mg; folic acid, 1 mg; Minerals: iron, 55 mg (as ferrous monohydrate) and 55 mg (as ferrous siderate); iodine, 0.6 mg (as anhydrous calcium iodide); Cu, 10 mg (as copper sulfate pentahydrate); manganese, 50 mg (as manganese oxide); Zn, 100 mg (as zinc oxide); Se, 1 mg (as sodium selenite); phytase OUT 250.

Before entering the farrowing unit (d-4) and at weaning (d27), the sows were weighed, and the muscle and backfat depths were measured by ultrasound (LS-1000, Tokyo Keiki Inc., Tokyo, Japan) at the P2 position (left side of the 10th rib and 6 cm from the sow’s backbone). At farrowing, the number of piglets born alive was recorded, and within 24 h, cross-fostering of piglets within sows in the same groups was performed. Cross-fostering was performed to standardize litter size and match the rearing capacity of the sow to ensure access to a functional teat for all piglets. Piglets were individually weighed immediately after farrowing (d0 before cross-fostering), at weaning (d27), and 14 d after weaning (d41) to calculate piglet and litter average daily gain **(ADG)**.

At farrowing, a subgroup of 8 sows per diet (balanced for parity and number of newborn piglets) was selected for colostrum and milk sampling (d10 and d20 of lactation). Samples were collected from all teats of the sows as reported by Luise et al. (2018) and stored at 4 °C for analysis of protein, fat, lactose, and urea concentrations and somatic cell count **(SCC)**.

At weaning, a fecal sample was collected from the same subset of sows. Briefly, the sows were gently stimulated, and fecal samples were collected into a sterile collection tube, immediately frozen in liquid nitrogen, and then stored at −80 °C until microbiome analysis. Furthermore, at weaning, 32 piglets (1 piglet per sow with an average BW within the litter) were taken to the experimental facility of the University of Bologna, where they were slaughtered. The transport lasted approximately 1 h; the piglets were not fasted prior to transport, and given the short duration of the journey, no refeeding was planned upon arrival. Once arrived, the piglets were housed in pens in pairs (2 piglets per group) and immediately processed for euthanasia, alternating between the two experimental groups to avoid any systematic bias. The piglets were deeply anesthetized with Pentothal Sodium (10 mg/kg BW, MSD Animal Health S.r.l., MI, Italy) and sacrificed by intracardiac injection of Tanax (0.5 mL/kg BW, MSD Animal Health S.r.l., MI, Italy) according to the Animal Research: Reporting of In Vivo Experiments (ARRIVE) guidelines. Immediately after slaughter, the intestine was excised, and the cecum was isolated and tied off at both ends to prevent content leakage. From the caudal section, approximately 5 g of cecal content was aseptically collected using sterile instruments and transferred into a sterile tube. Samples were snap frozen in liquid nitrogen and stored at −80°C.

After weaning, the piglets that were not slaughtered were moved to a commercial weaning unit. There, the pigs from both groups were mixed and housed in pens of 20 animals each. The post-weaning housing facility featured fully slatted concrete floors, as well as automated ventilation and temperature control. The ambient temperature started at 27 °C and was gradually decreased to approximately 21 °C as the pigs gained weight. Pigs had continuous access to feed and water. Piglets were weighed on day 41.

### Colostrum and milk analysis

Colostrum and milk composition for total fat, total protein, casein, lactose, urea, dry matter, and SCC were analyzed in triplicate using the Milkoscan FT2 infrared spectroscope (FOSS A/S, Padua, Italy).

### Bacterial DNA extraction and sequencing

Total bacterial DNA for microbiota analysis was extracted from fecal and cecal samples using a FastDNATM Spin Kit for Soil (MP Biomedicals, LLC, Santa Ana, CA, USA). DNA quantity and quality were assessed using a NanoDrop ND 1000 spectrophotometer (NanoDrop Technologies Inc., Wilmington, DE, USA). Next-generation sequencing **(NGS)** of bacterial DNA was performed as reported by Luise et al. (2021b). Library construction and 16S rRNA gene sequencing were performed using the MiSeq Reagent Kit V3-V4 on the MiSeq-Illumina platform.

### Virome DNA and RNA extraction and sequencing

To extract viral DNA and RNA, 10% fecal/cecal suspension was homogenized (Tissue rupture II, Qiagen) and centrifuged at 4 °C for 30 min at 9,000 rpm. The supernatant was filtered through a 0.45 µm Merck Millex Syringe Filter, Durapore (PVDF) to remove eukaryotic and bacterial cell-sized particles. The filtrate was then purified and concentrated in 100K Amicon tubes to a final volume of 140 to 200 µL. The concentrated supernatant was treated with DNAse, benzonase, and RNase (Thermo Fisher Scientific) at 37 °C for 90 min to digest unprotected nucleic acids. Viral nucleic acids were extracted using the AllPrep Power Viral DNA/RNA Kit (Qiagen). Viral RNA was reverse transcribed into complementary DNA **(cDNA)** using the Maxima H-minus double-stranded cDNA synthesis kit (Thermo Fisher Scientific). Amplified DNA/cDNA was purified using the GeneJet polymerase chain reaction **(PCR)** Purification Kit (Thermo Fisher Scientific). DNA was quantified using the double-stranded DNA (dsDNA) HS Assay Kit (ThermoFisher Scientific) on a Qubit 3.0 instrument (Life Technologies) and processed for labeling and indexing using the Illumina Nextera XT Library Prep Kit (Illumina, San Diego, CA, USA) according to the manufacturer’s protocol. Libraries were then diluted and sequenced on the Illumina Miseq platform using a V3-600 cycle chemistry.

### Bioinformatics and statistical analysis

Performance data were analyzed in R software version 4.1.1 (https://cran.r-project.org/bin/windows/base/old/4.1.1/) using the “car” and “lsmeans” packages. Performance data were analyzed using an ANOVA model with diet treatment (CO vs. ARG), batch, and parity order as fixed factors and litter size as covariates. A general linear model with a binomial distribution and a logit link was used for mortality data. Sows were used as the experimental unit.

Microbiota analysis for bacteria was performed using the DADA2 pipeline (Callahan et al., 2016) in R software version 4.1.1. Taxonomic categories were assigned using the Silva database (release 138) (Quast et al., 2013) as a reference. The alpha (Shannon, Chao1, and InvSimpson indices) and beta diversity (calculated as the Bray–Curtis distance matrix), and the abundance of taxonomic categories, were analyzed using R software with the “PhyloSeq” (McMurdie and Holmes, 2013) and “Vegan” (Dixon, 2003) packages. Alpha diversity indices were analyzed using an ANOVA model (lm function), including sample type (sows vs. piglets) and diet (CO vs. ARG), followed by pairwise comparisons. Beta diversity was analyzed using an Adonis test model (adonis.test function), including sample type (sows vs. piglets) and diet (CO vs. ARG) as factors. The effects on beta diversity were visualized using a nonmetric multidimensional scaling **(NMDS)** approach (plot_ordination function).

Differences in taxonomic abundance between sow and piglet bacterial taxa were analyzed using the DESeq2 package based on negative binomial generalized linear models, including and applying the Benjamini–Hochberg method for multiple testing correction (estimateSizeFactors function) (Love et al., 2013). Taxa with an adjusted *P*-value (false discovery rate **[FDR]**) less than 0.05 and an absolute log2 fold change greater than 0.5 were considered significant. Linear discriminant analysis effect size **(LefSe)** analysis at the genus level was applied to identify taxa differentially expressed (linear discriminant analysis **[LDA]** >3 and P.adj <0.05) between dietary treatment within sows and piglets bacterial microbiome (Segata et al., 2011).

For the virome, the raw sequencing reads were first trimmed and checked for quality using Trimmomatic (version 0.39). Ambiguous nucleotides (N’s), short reads (<36 nt), and low‐quality bases were trimmed with a sliding window size of 4. Paired-end clean reads were aligned to the Sus Scrofa reference genome (Sscrofa11.1.101) using Bowtie2 (v2.3.5.1) (Langmead and Salzberg, 2012) and Samtools (v.1.5) (Li et al., 2009), using the default parameters, to filter and remove host genome contamination. The remaining reads were *de novo* assembled using Spades (v3.14.0) (Bankevich et al., 2012), and contigs were compared to the National Center for Biotechnology Information **(NCBI)** nonredundant protein database (nr20200210 version) using Diamond BLASTx (v2.0.13) (Buchfink et al., 2014).

The taxonomic distribution of the classified reads was visualized using Krona (v.2.7.1) (Ondov et al., 2011). Differences in viral abundance between sows and piglets at weaning and accounting for feeding effects were quantified using BWA-MEM (Li, 2013) and further analyzed using DESeq2 and PhyloSeq (McMurdie and Holmes., 2013) and Vegan (Dixon, 2003) packages in R (v3.2.3).

Alpha diversity was assessed using the Shannon alpha diversity metric. Beta diversity was visualized using the NMDS plot with Bray–Curtis distances, and significance was calculated using PERMANOVA with the Adonis function from the Vegan package.

In addition, a Spearman correlation analysis was performed using the Hmisc package (v5.1-3) to further investigate the relationship between the virus and bacterial communities. The correlations were plotted using a heat map generated with the pheatmap package (v1.0.12).

Data were reported in the tables as estimated mean and SEM. Results were considered significant at *P* ≤ 0.05, and tendencies were observed at 0.05 < *P* ≤ 0.10.

## Results

### Performance, health, and milk composition

The results of sow and piglet growth performance are shown in Table 2. The diet did not affect the BW of the sows at weaning (*P* = 0.36), their ADG (*P* = 0.19), or the loss of muscle and backfat (*P* > 0.20). The diet did not affect the number of piglets born (*P* = 0.13) and weaned (*P* = 0.67), but supplementation with Arg tended to reduce the percentage of piglet mortality during the weaning period (*P* = 0.06) and up to 14 d post-weaning (d14; *P* = 0.08). The diet did not affect the piglets’ BW at birth (*P* = 0.33) and at weaning (*P* = 0.42) or their ADG at weaning (*P* = 0.20), but the ADG of piglets reared by sows fed Arg was higher from birth to 14 d post-weaning (*P* = 0.04).

**Table 2.** Effect of top-dressed arginine on sows and litters performance

Items	Diet^1^		SEM	***P*-value^2^** **Diet**
	CO	ARG		
	*n* = 16	*n* = 16		
Sow BW, d4, kg	310	310	8.03	0.96
Sow BW, d28, kg	258	248	8.14	0.39
Sow ADG, kg/d	−1.68	−1.98	0.17	0.19
Loss backfat, mm	5.48	6.34	0.53	0.24
Loss muscle, mm	2.68	3.58	0.60	0.37
*N* piglets at birth	14.20	13.30	0.44	0.13
*N* piglets at weaning	11.10	11.30	0.37	0.68
Mortality at weaning, %	2.08^a^	1.28^b^	0.30	0.06
Mortality at 14 d post-weaning %	2.16^a^	1.45^b^	0.20	0.08
Piglet BW at birth, kg	1.46	1.55	0.67	0.34
Piglet BW at weaning, kg	7.10	7.40	0.30	0.42
Piglet ADG at weaning kg/d	0.21	0.22	0.07	0.20
Piglet ADG at 14 d post-weaning, kg/d	0.22^B^	0.26^A^	0.01	0.04

1 Diets: CO = the group fed a standard lactating sow diet; Arg = the group fed the standard lactating sow diet plus 22.5 g/d/sow of L-Arg. Data are reported as average mean and SEM. n = number of litters per group.

2 Data were analyzed using a general linear model and ANOVA model with diet treatment (CO vs. ARG), batch, and parity order as fixed factors and litter size as covariates. A general linear model with a binomial distribution and logit link was used for mortality data.

a,b Within a row, values without common subscriptions differ with a 0.05 < *P* ≤ 0.10.

A,B Within a row, values without common subscriptions differ with a *P* ≤ 0.05.

Diet did not affect the proximal composition of colostrum (*P* > 0.10). At d10 of lactation, Arg supplementation significantly reduced the percentage of fat in milk (*P* = 0.05), while no effect was observed on the other components (*P* > 0.10). At d20 of lactation, Arg supplementation reduced the percentage of fat (*P* = 0.03) and tended to increase the percentage of lactose (*P* = 0.05) (Table 3).

**Table 3.** Effect of top-dressed arginine on sows’ colostrum and milk composition

Items	**Diet** ^1^		SEM	***P*-value** ^2^
	CO	ARG		
	*n* = 8	*n* = 8		Diet
Colostrum				
Fat, %	3.37	4.59	0.30	0.64
Lactose, %	3.52	3.39	0.16	0.58
Protein, %	20.0	21.2	1.10	0.45
Urea, %	45.0	47.1	1.48	0.35
SCC, *N* × 1,000/mL	597	412	110	0.26
Day 10, milk				
Fat, %	10.98^A^	7.76^B^	0.94	0.05
Lactose, %	5.44	5.17	0.28	0.53
Protein, %	7.29	7.31	0.30	0.95
Urea, %	44.2	40.0	2.66	0.37
SCC, *N* × 1,000/mL	757	1,158	643	0.66
Day 20, milk				
Fat, %	11.00^A^	7.70^B^	0.83	0.03
Lactose, %	5.40^B^	5.97^A^	0.17	0.05
Protein, %	7.29	7.31	0.30	0.95
Urea, %	44.10	40.50	2.71	0.38
SCC, *N* × 1,000/mL	721	1,216	649	0.61

1 Diets: CO = the group fed a standard lactating sow diet; Arg = the group fed a standard lactating sow diet plus 22.5 g/d/sow of L-Arg. Data are reported as average mean and SEM.

2 Data were analyzed using a general linear model and ANOVA model with diet treatment (CO vs. ARG), batch, and parity order as fixed factors and litter size as covariates.

A,B Within a row, values without common subscriptions differ with a *P* ≤ 0.05.

Abbreviation: SCC, somatic cell count.

### Bacterial profile

All fecal and cecal samples were subjected to DNA extraction and 16S rRNA gene sequencing. A total of 979,147 sequences were obtained from the samples of different animals sequenced individually. The average number of reads per sample after processing was 30,598.3. A total of 1,003 amplicon sequence variants **(ASVs)** were identified by clustering sequences at 97% sequence homology using the DADA2 pipeline (Callahan et al., 2016) and the Silva (v.138.1) database (Quast et al., 2013).

The bacterial profile of the sows was composed of 14 phyla, with Firmicutes (71.40%), Bacteroidota (19.59%), and Spirochaetota (5.51%) representing 96.49% of the total composition. At the family level, Peptostreptococcaceae (22.65%), Clostridiaceae (19.28%), and Rikenellaceae (8.04%) were the most abundant, and at the genus level, *Clostridium* sensu stricto 1 (19.28%), *Terrisporobacter*, and *Romboutsia* (7.17%) were the most abundant. The bacterial profile of piglets was composed of 16 phyla, with Firmicutes (84.87%), Bacteroidota (8.51%), and Proteobacteria (1.96%) representing 95.34% of the total composition. Considering the family level, Lactobacillaceae (26.37%), Lachnospiraceae (15.50%), and Ruminococcaceae (10.53%) were the most abundant, and considering the genus level, *Lactobacillus* (26.37%), *Subdoligranulum* (8.31%), and *Romboutsia* (5.61%) were the most abundant.

**Figure 1.**
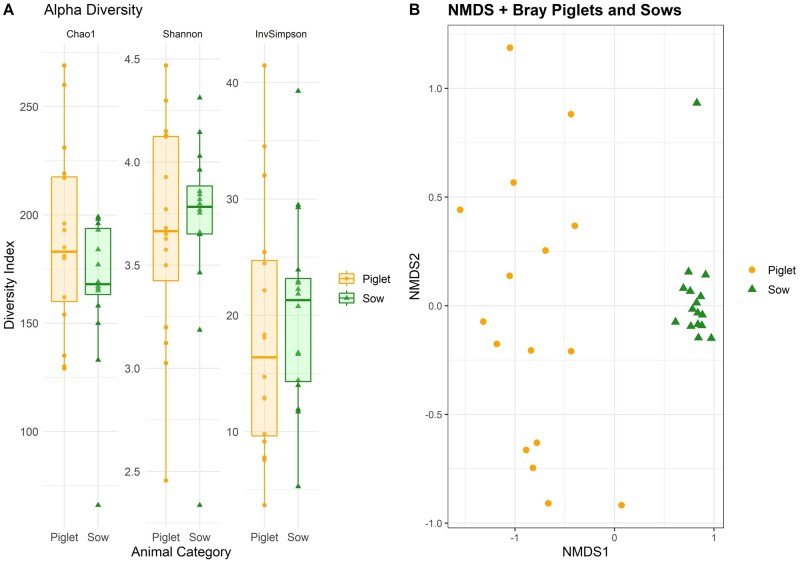
Alpha indices (A) and nonmetric multidimensional scaling plot (B) of the bacterial microbiome of piglets and sows at weaning.

Analysis of alpha diversity metrics using Shannon’s, Chao’s, and InvSimpson’s indices showed no differences between sows and piglets and between diet groups (Figure 1A; *P* > 0.10). To understand the differences between the fecal bacterial microbiota of sows and piglets, beta diversity was estimated. The Bray–Curtis distance was used to generate the beta diversity distance matrix and calculate the degree of differentiation between samples (Figure 1B). In agreement with the NMDS plot, betadisper analysis showed a significant difference in the dispersion between sow and piglet samples (*P* = 0.01), indicating that the bacterial community of piglets has a greater dispersion than that of sows. Sample dispersion was not affected by diet group (*P* = 0.48). Analysis of variance using the Adonis test showed a significant difference between sows and piglets (R^2^ = 0.36, *P* < 0.01) and no difference by dietary treatment (R^2^ = 0.02, *P* = 0.61).

The significant results for the taxonomic differences between sows and piglets are presented in Supplementary Table 1. At the phylum level, sows had a higher abundance of Spirochaetota, Patescibacteria, Thermoplasmatota, Bacteroidota, and Desulfobacterota (*P*.adj < 0.01), and piglets had a higher abundance of Fusobacteriota, Synergistota, Proteobacteria (*P*.adj < 0.01), Actinobacteriota (*P*.adj = 0.02), and Firmicutes (*P*.adj < 0.01). At the family level, 29 families differed significantly between sows and piglets; among them, sows had a higher abundance of Butyricicoccaceae, Bacteroidales p-2534-18B5 gut group, Spirochaetaceae, Clostridiaceae, and Rikenellaceae (*P*.adj. < 0.01), and piglets had a higher abundance of Streptococcaceae, Pasteurellaceae, Fusobacteriaceae, Defluvialeaceae, Enterobacteriaceae, Ruminococcaceae, and Lactobacillaceae (*P*.adj < 0.01). At the genus level, 78 genera differed between sows and piglets; among them, sows had a higher abundance of *Intestinibacter*, *Treponema*, Lachnospiraceae UCG-009, Prevotellaceae UCG-001, Lachnospiraceae AC2044 group, and Butyricicoccaceae UCG-008 (*P*.adj < 0.01), and piglets had a higher abundance of *Roseburia*, Lachnospiraceae CHKCI001, *Lachnoclostridium*, *Streptococcus*, *Negativibacillus*, *Actinobacillus*, *Subdoligranulum*, *Dorea*, and *Escherichia-Shigella* (*P*.adj < 0.01).

Considering the sows and piglets independently, the diet treatment did not affect the alpha diversity indices, the sample dispersion and the beta diversity index of the sows (*P* > 0.10). The diet significantly affected the abundance of specific bacterial taxa; the ARG group was characterized by a higher abundance of *Romboutsia* (LDA score = 4.25, *P*.adj = 0.05) and *Marvinbryantia* (LDA score = 3.09, *P*.adj = 0.04), whereas the CO group was characterized by a higher abundance of the taxa UCG-010 belonging to the Oscillospirales (LDA score = 3.41, *P*.adj < 0.01), UCG-002 belonging to the family of the Oscillospiraceae (LDA score = 3.34, *P*.adj = 0.03), RF39 belonging to the Bacilli (LDA score = 3.06, *P*.adj = 0.02), and *Anaerovibrio* (LDA score = 3.06, *P*.adj = 0.05).

**Figure 2.**
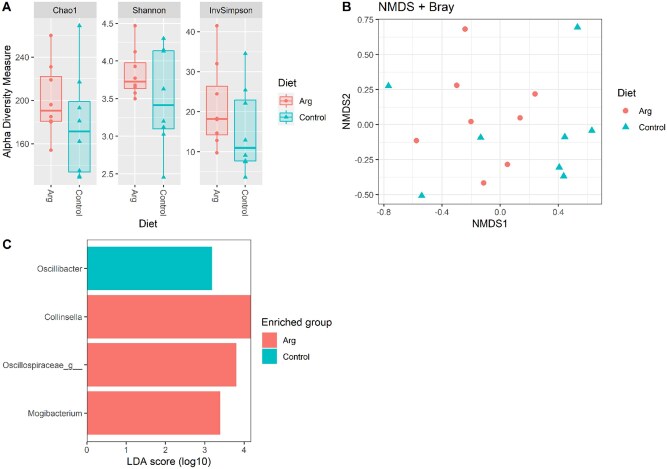
Effect of feeding sows with top-dressed arginine on alpha diversity (A), beta diversity (B), and taxa abundance (C) of cecal samples of their piglets at weaning. The Shannon index tended to increase (*P* = 0.08) in piglets from sows receiving arginine.

Supplementing maternal diet with Arg tended to increase the Shannon index of piglet bacterial microbiota (*P* = 0.08) (Figure 2A), while beta diversity was not affected by maternal diet (Figure 2B; *P* = 0.68). The dam’s diet significantly affected the abundance of specific bacterial taxa of piglets; the ARG group was characterized by a higher abundance of *Collinsella* (LDA score = 4.16, *P*.adj = 0.05), an unknown genus belonging to the family Oscillospiraceae (LDA score = 3.78, *P*.adj = 0.03), and *Mogibacterium* (LDA score = 3.39, *P*.adj = 0.05), while the CO group was characterized by *Oscillibacter* (LDA score = 3.19, *P*.adj < 0.01) (Figure 2C).

### Virome profile

The viral profile of sows was composed of 11 orders, with Petitvirales (82.34%), Caudovirales (12.89%), and Cremevirales (1.46%) being the most abundant. The viral profile of piglets was composed of 10 orders, with Caudovirales (65.88%), Petitvirales (29.93%), and Tubulavirales (1.23%) being the most abundant.

**Figure 3.**
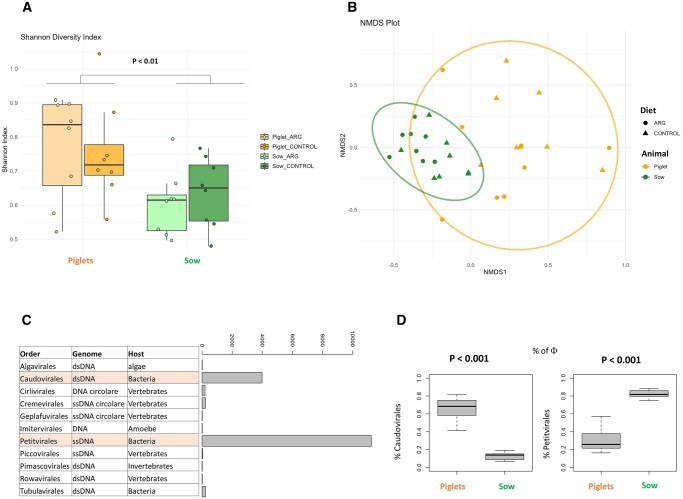
Alpha indices (A) and nonmetric multidimensional scaling plot (B) and difference in order abundance (C and D) between the viral microbiome of piglets and sows at weaning.

The analysis of alpha diversity metrics using the Shannon index showed a significantly higher value in piglets compared to sows (*P* < 0.01) (Figure 3A). Furthermore, considering the beta diversity, the viral profile of sows and piglets differed significantly (*P* < 0.01); in fact, the samples were plotted in an NMDS plot clustered according to the age class (Figure 3B). The results for the taxonomic differences between sows and piglets are presented in Supplementary Table 2. The sows had a lower relative abundance of Caudovirales (*P* < 0.01) and a higher abundance of Petitvirales (*P* < 0.01) compared to the piglets (Figure 3C and D).

**Figure 4.**
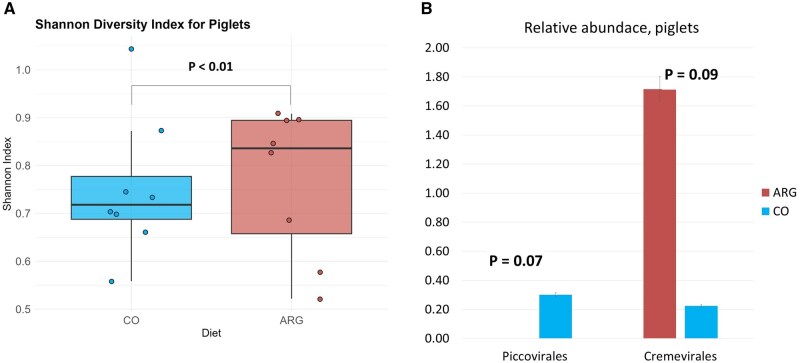
Effect of feeding sows top-dressed arginine on alpha diversity (A) and viral order abundance (B) of cecal samples of their piglets at weaning.

Considering the effect of sow diet on the viral profile of sows and piglets, no difference in alpha diversity and order abundance was observed for sows (*P* > 0.10). The dam’s diet significantly affected the Shannon index of the viral profile of the piglets; the Shannon index was higher in the piglets of the ARG group (*P* < 0.01) (Figure 4A). No difference in the abundance of viral taxa was observed in the sows (*P* > 0.10), while the diet of the dam tended to influence some viral taxa in her offspring. The ARG group tended to have a lower abundance of Piccovirales (*P* = 0.07) and a higher abundance of Cremevirales (*P* = 0.09) (Figure 4B).

## Correlation between bacterial and viral profile

**Figure 5.**
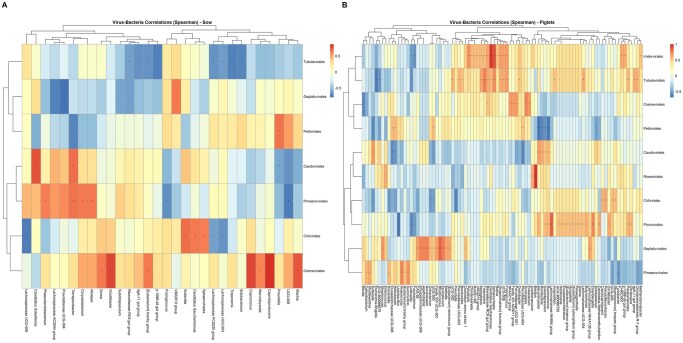
Heatmap of Spearman correlation analysis between viral orders and bacterial genera in sows’ feces (A) and piglets’ cecal content (B). Each cell represents the Spearman correlation coefficient (r) between a viral order and a bacterial genus. Color intensity indicates the strength and direction of the correlation, ranging from deep blue (strong negative correlation) to deep red (strong positive correlation). Asterisks indicate significance levels (*P < 0.05 and **P < 0.01).

To investigate the relationship between the bacterial and viral communities in the sows and piglets, Spearman correlations were performed, and the results are shown in Figure 5. For the sows, the strongest positive correlations were observed between the viral order Cremevirales and bacterial genera *Caproiciproducens* (r = 0.86; *P* < 0.01), *Oscillibacter* (r = 0.80; *P* < 0.01), *Coprococcus* (r = 0.79; *P* < 0.01), and *Slackia* (r = 0.73; *P* = 0.01) and between the viral order Caudovirales and the bacterial genera *Candidatus soleaferrea* (r = 0.79; *P* < 0.01). Conversely, the strongest negative correlations were between the viral order Geplafuvirales with the bacterial genera Prevotellaceae UCG-004 (r = −0.79; *P* < 0.01) and Lachnospiraceae AC2044 group (r = −0.73; *P* = 0.01), the viral order Pimascovirales with the bacteria genus *Butyricicoccaceae* UCG-008 (r = −0.75; *P* < 0.01), the viral order Cirlivirales with the genus Lachnospiraceae UCG-009 (r = −0.75; *P* < 0.01), and the viral order Tubulavirales with the bacterial genus *Treponema* (r = −0.75; *P* = 0.01). For the piglets, the strongest positive correlations were between the viral order Caudovirales with the bacterial genera *Sutterella* (r = 0.68; *P* < 0.01), *Marvinbryantia* (r = 0.65; *P* < 0.01)*, Romboutsia* (r = 0.60; *P* = 0.02), and *Ruminococcus* (r = 0.57; *P* = 0.03), and of the viral order Petitvirales with the bacterial genera *Helicobacter* (r = 0.55; *P* = 0.03) and *Erysipelotrichaceae* UCG-006 (r = 0.55; *P* = 0.04); while the strongest negative stronger negative correlations were between the viral order Petitvirales with the bacterial genera *Marvinbryantia* (r = −0.69; *P* < 0.01), *Ruminococcus* (r = −0.65; *P* < 0.01), *Romboutsia* (r = −0.62; *P* = 0.01) and *Sutterella* (r = −0.60; *P* = 0.02), and of the viral order Caudovirales with the bacterial genus *Prevotellaceae* UCG-004 (r = −0.60; *P* = 0.01).

## Discussion

In recent years, research on the gut microbiota of sows and their offspring has increased, following the establishment of a link between animal health and their microbiota. The recognition of microbial transmission between sows and their offspring has emerged as a critical element in piglet microbiota development (Hou et al., 2015). One of the key factors that regulates this distinct mother-offspring ecosystem in pigs is the piglet’s close interaction with the maternal microbiota during parturition and lactation, particularly through fecal samples (Leblois et al., 2018; Chen et al., 2022; Palumbo et al., 2023). However, most literature on the gut microbiota is limited to the description of the bacterial community, neglecting the viral and fungal communities. The study clearly demonstrated the differentiation of bacterial and viral populations between sows and their piglets, as well as the interplay between these two microbiological populations.

The sow’s bacterial population exhibited less dispersion in terms of beta diversity than her piglets. This finding is consistent with previous research indicating that piglet bacterial populations are less mature and stable compared to those of adult pigs, such as sows, and therefore may exhibit greater dispersion (Luise et al., 2021b). The viral population exhibited a similar distribution pattern to that of bacteria. Compared to piglets, the viral community of sows exhibited less dispersion (beta diversity). Interestingly, sows showed also a lower alpha diversity than piglets. While this is contrary to what is typically observed in humans, previous longitudinal studies in pigs have shown that viral species richness increases shortly after birth and subsequently tends to decrease and stabilize with age (Chen et al., 2023). Thus, our findings are consistent with the pig-specific pattern of temporal viral community dynamics. This result can be explained by the maturation process: in piglets, colonization is still ongoing, resulting in a transiently higher species richness and greater inter-individual variability. With increasing age, the viral community tends to stabilize, with fewer dominant species and more homogeneous profiles across animals, as observed in sows.

Furthermore, it is noteworthy that the viral community present in piglets had a higher abundance of the order Caudovirales, while in sows, the order Petitvirales was the most abundant. These orders are commonly found in the feces and intestinal contents of pigs (Billaud et al., 2021; Tao et al., 2022; Chen et al., 2023; Ji et al., 2023). In addition, Billaud et al. (2021) observed results similar to those of our study, noting that piglets had significantly higher abundances of Caudovirales than adult pigs. Caudovirales have also been associated with an unhealthy gut and a higher prevalence of diarrhea in both piglets and humans (Liu and Stappenbeck, 2016; Ji et al., 2023), which is consistent with piglets’ less mature guts compared to those of sows. Furthermore, it is noteworthy that both Caudovirales and Petitvirales are bacteriophages. The fact that these two orders differ between sows and piglets suggests that they play a crucial role in regulating the microbial community at the two different ages of the animal. Indeed, it is known that, in some animal species, gut bacteriophages can exert significant selective pressure on some resident bacterial populations. Phages may play an important role in self-regulating the microbial community and maintaining bacterial diversity (Letarov and Kulikov, 2009). The present study offers an intriguing finding: the correlations performed between viruses and bacteria in sows and piglets confirm the tendency for certain viral orders to co-occur with specific bacterial genera that are particularly abundant in each animal group. For instance, the viral order Caudovirales, which exhibited higher relative abundance in piglets, demonstrated a positive correlation with the bacterial genera *Subdoligranulum*, *Ruminococcus*, *Romboutsia*, and *Marvinbryantia*. According to our analyses, the relative abundance of these genera was higher in piglets than in sows. Furthermore, these genera were among the most abundant genera overall in piglet samples.

Conversely, the correlation between the relative abundance of the viral order Piccovirales and the bacterial genera that exhibited a positive correlation with it in sows is less evident. A positive correlation was observed between Piccovirales and *Prevotella*, with the former being more prevalent in piglets (whereas Prevotellaceae UCG-001 was more abundant in sows), and a negative correlation was identified between Piccovirales and the genus *Terrisporobacter*, which was, in contrast, more abundant in sows.

To the best of our knowledge, no other studies have investigated these specific relationships between viral orders and bacterial genera in pigs. In other species, virus–bacterium interactions are typically reported at the phylum level or in disease contexts, making them not directly comparable to the findings of this study (Sausset et al., 2020; Shah et al., 2023). Consequently, it is challenging to draw definitive conclusions, and this aspect should be further explored in future studies. Ideally, this exploration would involve increasing sequencing depth for both viruses and bacteria to understand their potential relationships.

Diet is widely recognized as an important factor that can influence microbial composition, serving as a powerful tool for its modulation. Previous studies have suggested that Arg has a limited but present effect in modulating the gut microbiota of pregnant and lactating sows (Luise et al., 2020; Luise et al., 2023; Virdis et al., 2024). Consistent with previous findings, Arg supplementation during lactation did not significantly alter the bacterial community of sows at weaning. In addition, the current study indicates that supplemental Arg had no effect on the viral community present in the fecal samples of the sows. This is the first study to investigate the effects of Arg within the viral community in samples of porcine intestinal content. Therefore, no direct comparisons with existing literature can be made, and it remains unclear whether the observed effects are due to the timing of Arg administration, the dosage used, or both. This study only investigated one dose and duration of administration; however, it is reasonable to assume that Arg does not negatively impact the microbial community of sows. In contrast, Arg supplementation affected the microbial community of their offspring. This may be related to the greater plasticity of the immature microbiota in piglets, which is more responsive to dietary and environmental changes compared to the stable and resilient community of sows. Early life, indeed, represents a key “window” for microbial manipulation, characterized by priority effects and niche preemption, which make the community particularly sensitive to external factors and may facilitate detectable changes in piglets but not in adult animals (Trevisi et al., 2021; Quan et al., 2023). Specifically, Arg supplementation tended to increase the Shannon index of the bacterial community and significantly increased that of the viral community in the offspring. A higher Shannon index corresponded to an increase in gut eubiosis, especially in early life stages, as functional redundancy and commensalism among microorganisms could serve as an alternative to competition, leading to greater fitness among the different microbial groups that make up the gut microbiota (Foster et al., 2017). Regarding the effect of Arg on the viral community, its supplementation tended to promote the growth of Cremvirales while reducing that of Piccovirales. The Piccovirales comprise robust, nonenveloped T = 1 icosahedral virus that are 21 to 22 nm in diameter. The virion is composed of 60 copies of the VP protein, and Piccovirales are among the smallest known viruses, along with circoviruses. The order Piccovirales includes the family Parvoviridae, which currently includes three subfamilies: Densovirinae, which infect arthropods; Parvovirinae, which infect vertebrates; and Hamaparvovirinae, which was recently established (Pumpens & Pushko, 2022). Porcine parvovirus **(PPV)** is a common virus in pigs that is typically asymptomatic and ubiquitous in global pig populations. However, in certain cases of infection, it can cause SMEDI syndrome, which includes stillbirth, mummification, embryonic death, and infertility. The order Cremevirales comprises 12 genera and 1 family (Smacoviridae), including the porprismacoviruses, which have been found in a variety of animals, including pigs (Fehér et al., 2021). However, the sequencing performed in this study was unable to identify the species; therefore, it is not possible to speculate that Arg supplementation decreased the abundance of plum pox virus of a specific species belonging to the order Cremevirales. Further studies are needed.

Regarding the bacterial community, the Arg supplementation promoted the growth of three bacterial genera in piglets: *Collinsenella*, *Mogibacterium*, and some genera within the Oscillospiraceae family. Notably, *Collinsenella* and *Mogibacterium* have been identified in the microbiota of young piglets and have been shown to increase with probiotic supplementation, including *Saccharomyces cerevisiae* (Kiros et al., 2018; Kiros et al., 2019). Furthermore, *Collinsenella* has been identified as a significant user of lactose in the human gut (Bag et al., 2017), which is supported by the higher abundance of lactose in milk at d20 of sows from the ARG group. This likely explains the increased abundance of this taxon in the cecal content of their offspring. More generally, these results suggest that the changes observed in the piglet cecal microbiota may be indirectly mediated by modifications in milk composition induced by maternal Arg supplementation. This is consistent with previous studies (Palumbo et al., 2024; Lee et al., 2024) showing that dietary interventions in sows can alter the biochemical composition of colostrum and milk, which in turn can influence the gut microbiota of their offspring. The mechanisms underlying these changes may involve multiple physiological effects of Arg in the lactating sow (Cruz et al., 2025). Arg supplementation can enhance the production of nitric oxide and polyamines, promoting cell proliferation and vasodilation, which potentially increases blood flow to the mammary gland and improves nutrient delivery for milk synthesis and protein production in mammary epithelial cells, thereby supporting lactogenesis in sows (Mateo et al., 2008; Ma et al., 2018; Luise et al., 2023). Furthermore, Arg may exert a secretagogue effect on hormones such as insulin, prolactin, and growth hormone, which may enhance milk yield and modify its biochemical composition, including lactose content (Wu et al., 2009; Zhu et al., 2017; Cruz et al., 2025). However, since these physiological parameters were not analyzed in the present study, direct links between maternal Arg supplementation, specific changes in milk composition, and piglet microbiota remain hypothetical and require further investigation.

Finally, Arg supplementation contributed to increased ADG of piglets and tended to reduce their mortality in the post-weaning phase. Weaning is generally known as one of the most critical periods for piglets, leading to loss of intestinal microbial eubiosis, impaired growth performance, and consistent mortality (Gresse et al., 2017; Luise et al., 2021a). The fact that piglets reared by sows receiving the Arg had better performance and lower mortality after weaning could be partly explained by microbiota modulation and partly by the effect of Arg on milk production. Indeed, as mentioned before, the Arg supplementation increased the lactose concentration in milk at d20, which has been associated with increased milk yield in several studies (Beyer et al., 2007; Craig et al., 2019). This is particularly important as the lactation curve is already in a declining phase at d20 (Hansen et al., 2012). This result may indicate a lesser reduction in milk production with Arg administration and support the hypothesis that Arg can positively regulate the sows’ lactogenesis (Cruz et al., 2025).

## Conclusions

In conclusion, the present study confirms the interest of Arg for lactating sows to promote piglet growth and intestinal eubiosis. Furthermore, the study underscores the potential indirect impact of Arg supplementation in sows on shaping the microbiota of their offspring. Finally, the results between the viral and bacterial communities highlight the strong interplay between these two kingdoms, which appears to be modulated by bacteriophages. This aspect warrants further exploration.

## Supplementary Material

skaf319_Supplementary_Data

## Abbreviations:

AAs: amino acids
ADG: average daily gain
Arg: arginine
ARG: group fed with 22g/d/sows or arginine
ARRIVE: Animal Research: Reporting of In Vivo Experiments
ASVs: amplicon sequence variants
BW: body weight
cDNA: complimentary DNA
CO: control group
d: day
dsDNA: double-stranded DNA
EU: European Union
FDR: false discovery rate
LDA: linear discriminant analysis
LefSe: linear discriminant analysis effect size
NCBI: National Center for Biotechnology Information
NGS: next-generation sequencing
NMDS: the nonmetric multidimensional scaling
PCR: polymerase chain reaction
PPV: porcine parvovirus
SCC: somatic cells count
SRA: sequence read archive
