# Supplementary material for: On-top arginine supplementation during lactation affects milk composition, performance, and intestinal bacterial and viral microbial community of sows and their piglets
Source: J Anim Sci. 2025 Sep 16;103:skaf319. doi: 10.1093/jas/skaf319 (PMC12552097; doi:10.1093/jas/skaf319)
Supplement: skaf319_Supplementary_Data [file skaf319_supplementary_data.docx]

**Supplementary Table 1.** Different bacterial taxa between sows and piglets at weaning.

| **Taxa** | **baseMean^1^** | **log2FoldChange^2^** | **lfcSE^3^** | **pvalue^4^** | **padj^5^** | **Piglet^6^** | **Sow^7^** |
| --- | --- | --- | --- | --- | --- | --- | --- |
| Phylum level | | | | | | | |
| Fusobacteriota | 33.3 | -8.66 | 1.19 | <0.01 | <0.01 | 281.67 | 0.00 |
| Synergistota | 46.29 | -5.11 | 1.15 | <0.01 | <0.01 | 275.12 | 2.61 |
| Proteobacteria | 492.6 | -4.81 | 0.6 | <0.01 | <0.01 | 951.42 | 33.77 |
| Actinobacteriota | 255.94 | -1.4 | 0.54 | 0.01 | 0.02 | 806.76 | 140.70 |
| Firmicutes | 27883.37 | -1.27 | 0.25 | <0.01 | <0.01 | 39407.58 | 16359.16 |
| Desulfobacterota | 123.95 | -1.04 | 0.48 | 0.03 | 0.05 | 166.74 | 81.15 |
| Bacteroidota | 3321.85 | 0.84 | 0.24 | <0.01 | <0.01 | 2384.35 | 4259.34 |
| Thermoplasmatota | 7.23 | 2.99 | 0.83 | <0.01 | <0.01 | 1.48 | 12.97 |
| Patescibacteria | 22.61 | 4.79 | 0.73 | <0.01 | <0.01 | 1.58 | 43.65 |
| Spirochaetota | 638.89 | 4.89 | 0.93 | <0.01 | <0.01 | 41.62 | 1236.17 |
| Family level | | | | | | | |
| Streptococcaceae | 644.54 | -11.23 | 0.96 | <0.01 | <0.01 | 1288.62 | 0.46 |
| Pasteurellaceae | 32.82 | -8.4 | 1.35 | <0.01 | <0.01 | 130.13 | 0.00 |
| Fusobacteriaceae | 30.45 | -8.29 | 1.23 | <0.01 | <0.01 | 204.89 | 0.00 |
| Defluviitaleaceae | 17.48 | -7.49 | 1.19 | <0.01 | <0.01 | 34.96 | 0.00 |
| Enterobacteriaceae | 201.7 | -5.77 | 0.78 | <0.01 | <0.01 | 396.21 | 7.19 |
| Micrococcaceae | 5.23 | -5.75 | 1.86 | <0.01 | 0.01 | 10.46 | 0.00 |
| Hydrogenoanaerobacterium | 2.94 | -4.92 | 2.09 | 0.02 | 0.04 | 5.88 | 0.00 |
| Veillonellaceae | 97.02 | -4.79 | 1.52 | <0.01 | <0.01 | 187.31 | 6.73 |
| Bacteroidaceae | 244.44 | -4.47 | 0.76 | <0.01 | <0.01 | 467.84 | 21.03 |
| Corynebacteriaceae | 7.53 | -4.39 | 1.59 | 0.01 | 0.01 | 14.42 | 0.64 |
| Synergistaceae | 33.29 | -4.35 | 1.13 | <0.01 | <0.01 | 145.82 | 3.09 |
| Ruminococcaceae | 2256.48 | -4.19 | 0.51 | <0.01 | <0.01 | 4278.51 | 234.45 |
| Lactobacillaceae | 6875.3 | -3.02 | 0.81 | <0.01 | <0.01 | 12238.68 | 1511.93 |
| [Eubacterium] coprostanoligenes group | 554.72 | -2.71 | 0.31 | <0.01 | <0.01 | 963.05 | 146.39 |
| Lachnospiraceae | 2813.11 | -2.45 | 0.2 | <0.01 | <0.01 | 4754.50 | 871.73 |
| Acidaminococcaceae | 201.77 | -0.76 | 0.29 | 0.01 | 0.02 | 253.58 | 149.96 |
| Oscillospiraceae | 1500.9 | -0.55 | 0.2 | 0.01 | 0.01 | 1784.50 | 1217.30 |
| Peptostreptococcaceae | 4774.93 | 1.17 | 0.3 | <0.01 | <0.01 | 2931.73 | 6618.13 |
| Christensenellaceae | 795.77 | 1.2 | 0.49 | 0.01 | 0.03 | 481.71 | 1109.83 |
| Rikenellaceae | 1238.4 | 2.26 | 0.57 | <0.01 | <0.01 | 427.87 | 2048.93 |
| Methanomethylophilaceae | 8.9 | 2.96 | 0.92 | <0.01 | <0.01 | 2.05 | 15.76 |
| Clostridiaceae | 2765.41 | 3.23 | 0.39 | <0.01 | <0.01 | 533.12 | 4997.71 |
| Bacteroidales_ F082 | 396.36 | 4.45 | 1.19 | <0.01 | <0.01 | 34.62 | 758.09 |
| Paludibacteraceae | 15.22 | 4.46 | 1.03 | <0.01 | <0.01 | 1.24 | 29.20 |
| Spirochaetaceae | 700.75 | 4.61 | 1 | <0.01 | <0.01 | 55.16 | 1346.34 |
| Saccharimonadaceae | 17.17 | 5.01 | 0.73 | <0.01 | <0.01 | 0.98 | 105.32 |
| Bacteroidales p-2534-18B5 gut group | 364.53 | 5.57 | 1.03 | <0.01 | <0.01 | 64.26 | 714.04 |
| Butyricicoccaceae | 170.94 | 5.97 | 0.53 | <0.01 | <0.01 | 5.05 | 336.84 |
| Genus level | | | | | | | |
| *Roseburia* | 17.27 | -24.09 | 2.93 | <0.01 | <0.01 | 164.04 | 0.00 |
| Lachnospiraceae CHKCI001 | 593.28 | -13.23 | 1.24 | <0.01 | <0.01 | 1186.57 | 0.00 |
| *Lachnoclostridium* | 429.47 | -12.76 | 0.84 | <0.01 | <0.01 | 858.95 | 0.00 |
| *Streptococcus* | 899.7 | -12.06 | 0.97 | <0.01 | <0.01 | 1798.89 | 0.51 |
| *Negativibacillus* | 113.6 | -10.84 | 1.57 | <0.01 | <0.01 | 227.20 | 0.00 |
| *Actinobacillus* | 85.38 | -10.43 | 1.54 | <0.01 | <0.01 | 170.75 | 0.00 |
| [*Ruminococcus*] torques group | 75.64 | -10.26 | 1.23 | <0.01 | <0.01 | 151.28 | 0.00 |
| *Tuzzerella* | 64.67 | -10.03 | 1.07 | <0.01 | <0.01 | 129.34 | 0.00 |
| *Subdoligranulum* | 3234.89 | -9.79 | 0.81 | <0.01 | <0.01 | 6462.53 | 7.25 |
| *Cloacibacillus* | 36 | -9.49 | 1.72 | <0.01 | <0.01 | 71.92 | 0.09 |
| *Fusobacterium* | 41.89 | -9.4 | 1.25 | <0.01 | <0.01 | 341.80 | 0.00 |
| *Bilophila* | 29.39 | -8.89 | 1.21 | <0.01 | <0.01 | 58.77 | 0.00 |
| Defluviitaleaceae UCG-011 | 29.12 | -8.88 | 1.2 | <0.01 | <0.01 | 58.24 | 0.00 |
| [Eubacterium] fissicatena group | 26.8 | -8.76 | 1.44 | <0.01 | <0.01 | 53.61 | 0.00 |
| *Mogibacterium* | 57.25 | -8.4 | 1.59 | <0.01 | <0.01 | 114.21 | 0.28 |
| *Dorea* | 1003.87 | -7.92 | 0.91 | <0.01 | <0.01 | 1999.60 | 8.14 |
| Lachnospiraceae 44679 | 14.88 | -7.92 | 1.85 | <0.01 | <0.01 | 29.76 | 0.00 |
| Lachnospiraceae CAG-56 | 14.84 | -7.91 | 2.04 | <0.01 | <0.01 | 29.67 | 0.00 |
| *Intestinimonas* | 73.09 | -7.5 | 1.03 | <0.01 | <0.01 | 145.35 | 0.83 |
| *Anaerotruncus* | 10.89 | -7.45 | 1.68 | <0.01 | <0.01 | 21.78 | 0.00 |
| Lachnospiraceae GCA-900066575 | 9.2 | -7.22 | 1.29 | <0.01 | <0.01 | 18.40 | 0.00 |
| *Bifidobacterium* | 7.61 | -6.94 | 2.93 | <0.01 | 0.03 | 166.53 | 0.00 |
| *Alistipes* | 9.68 | -6.93 | 1.42 | <0.01 | <0.01 | 19.28 | 0.08 |
| *Escherichia-Shigella* | 261.81 | -6.82 | 0.76 | <0.01 | <0.01 | 519.11 | 4.52 |
| *Odoribacter* | 6.69 | -6.76 | 2.67 | <0.01 | 0.02 | 71.70 | 0.00 |
| Lachnospiraceae UCG-010 | 66.67 | -6.48 | 1.11 | <0.01 | <0.01 | 131.93 | 1.41 |
| Oscillospiraceae UCG-003 | 4.63 | -6.22 | 2.67 | <0.01 | 0.03 | 9.27 | 0.00 |
| *Corynebacterium* | 13.94 | -6.18 | 1.59 | <0.01 | <0.01 | 27.57 | 0.32 |
| *Butyricimonas* | 4.21 | -6.09 | 2.68 | 0.02 | 0.04 | 8.42 | 0.00 |
| *Fournierella* | 5.89 | -5.81 | 1.13 | <0.01 | <0.01 | 11.61 | 0.16 |
| *Megasphaera* | 47.52 | -5.68 | 1.71 | <0.01 | <0.01 | 223.15 | 3.96 |
| *Bacteroides* | 346.62 | -5.65 | 0.75 | <0.01 | <0.01 | 679.82 | 13.43 |
| *Synergistes* | 2.88 | -5.55 | 2.3 | 0.02 | 0.03 | 5.75 | 0.00 |
| *Ruminococcus* | 323.48 | -5.5 | 0.97 | <0.01 | <0.01 | 633.05 | 13.91 |
| *Howardella* | 3.64 | -5.27 | 1.9 | 0.01 | 0.01 | 7.08 | 0.19 |
| Lachnospiraceae UCG-004 | 25.39 | -4.79 | 1.77 | 0.01 | 0.01 | 49.03 | 1.75 |
| *Pyramidobacter* | 16.92 | -4.68 | 1.3 | <0.01 | <0.01 | 162.08 | 1.18 |
| *Alloprevotella* | 236.95 | -4.27 | 0.77 | <0.01 | <0.01 | 450.58 | 23.31 |
| *Lactobacillus* | 11993.4 | -4.18 | 0.86 | <0.01 | <0.01 | 22735.02 | 1251.79 |
| *Prevotella* | 137.05 | -3.97 | 1.24 | <0.01 | <0.01 | 543.15 | 16.37 |
| *Anaerovibrio* | 68.34 | -3.6 | 1.54 | 0.02 | 0.03 | 126.32 | 10.36 |
| *Holdemanella* | 169.34 | -3.56 | 0.62 | <0.01 | <0.01 | 312.30 | 26.39 |
| *Blautia* | 272.82 | -3.55 | 0.49 | <0.01 | <0.01 | 503.10 | 42.54 |
| Oscillospiraceae UCG-002 | 230.41 | -3.47 | 1.06 | <0.01 | <0.01 | 421.99 | 38.82 |
| *Coprococcus* | 519.06 | -3.4 | 0.35 | <0.01 | <0.01 | 948.63 | 89.49 |
| *Colidextribacter* | 155.95 | -3.36 | 0.58 | <0.01 | <0.01 | 284.18 | 27.72 |
| Oscillospiraceae UCG-005 | 553.59 | -2.61 | 0.57 | <0.01 | <0.01 | 951.39 | 155.79 |
| *Catenisphaera* | 155.06 | -2.5 | 0.65 | <0.01 | <0.01 | 622.07 | 46.35 |
| *Oscillibacter* | 30.64 | -2.3 | 0.88 | 0.01 | 0.02 | 51.07 | 10.22 |
| *Phascolarctobacterium* | 277.15 | -2.23 | 0.36 | <0.01 | <0.01 | 456.80 | 97.49 |
| *Marvinbryantia* | 68.49 | -2.13 | 0.61 | <0.01 | <0.01 | 111.71 | 25.26 |
| [Eubacterium] hallii group | 70.46 | -2.1 | 0.7 | <0.01 | 0.01 | 114.33 | 26.58 |
| *Oscillospira* | 159.75 | -2.08 | 0.53 | <0.01 | <0.01 | 258.64 | 60.85 |
| *Collinsella* | 104.09 | -1.95 | 0.74 | 0.01 | 0.02 | 520.06 | 42.61 |
| *Desulfovibrio* | 120.94 | -1.82 | 0.59 | <0.01 | <0.01 | 188.63 | 53.24 |
| *Parabacteroides* | 143.39 | -1.67 | 0.53 | <0.01 | <0.01 | 218.19 | 426.57 |
| *Romboutsia* | 2197.16 | -1.43 | 0.3 | <0.01 | <0.01 | 3206.37 | 1187.95 |
| *Turicibacter* | 464.79 | 0.77 | 0.34 | 0.02 | 0.04 | 430.61 | 2647.26 |
| *Terrisporobacter* | 2340.1 | 1.28 | 0.36 | <0.01 | <0.01 | 1364.39 | 3315.81 |
| Rikenellaceae RC9 gut group | 748.25 | 1.4 | 0.54 | 0.01 | 0.02 | 410.28 | 1086.21 |
| Candidatus Soleaferrea | 51.21 | 1.84 | 0.66 | 0.01 | 0.01 | 22.44 | 79.97 |
| *Clostridium* sensu stricto 1 | 1957.4 | 1.94 | 0.38 | <0.01 | <0.01 | 810.81 | 3103.98 |
| *Frisingicoccus* | 42.74 | 2.05 | 0.9 | 0.02 | 0.04 | 16.39 | 69.08 |
| [Anaerorhabdus] furcosa group | 16.15 | 2.13 | 0.78 | 0.01 | 0.01 | 5.81 | 26.50 |
| *Acetitomaculum* | 4.79 | 2.22 | 0.89 | 0.01 | 0.02 | 1.64 | 7.94 |
| Methanomethylophilaceae RumEn M2 | 5.24 | 2.65 | 0.87 | <0.01 | 0.01 | 1.23 | 9.25 |
| *Sphaerochaeta* | 16.72 | 2.86 | 0.72 | <0.01 | <0.01 | 10.80 | 29.67 |
| Rikenellaceae dgA-11 gut group | 103.52 | 3.19 | 0.77 | <0.01 | <0.01 | 20.76 | 186.29 |
| Oscillospiraceae V9D2013 group | 3.35 | 3.29 | 1.52 | 0.03 | 0.05 | 0.42 | 6.28 |
| Candidatus Saccharimonas | 10.59 | 3.62 | 0.73 | <0.01 | <0.01 | 1.46 | 85.47 |
| Butyricicoccaceae UCG-009 | 34.24 | 3.97 | 0.76 | <0.01 | <0.01 | 3.99 | 64.49 |
| Lachnospiraceae UCG-001 | 16.04 | 4.01 | 0.9 | <0.01 | <0.01 | 1.77 | 30.31 |
| *Intestinibacter* | 21.27 | 4.04 | 0.81 | <0.01 | <0.01 | 6.33 | 40.36 |
| *Treponema* | 452.02 | 4.45 | 0.92 | <0.01 | <0.01 | 39.24 | 864.80 |
| Lachnospiraceae UCG-009 | 44.7 | 4.6 | 1.02 | <0.01 | <0.01 | 3.49 | 85.91 |
| Prevotellaceae UCG-001 | 139.79 | 5.17 | 0.93 | <0.01 | <0.01 | 7.44 | 272.14 |
| Lachnospiraceae AC2044 group | 48.54 | 5.65 | 0.88 | <0.01 | <0.01 | 9.75 | 95.17 |
| Butyricicoccaceae UCG-008 | 86.95 | 6.42 | 0.92 | <0.01 | <0.01 | 1.89 | 172.00 |

baseMean^1^ = mean of normalized taxa counts averaged over all samples from both conditions. log2FoldChange^2^ = log2 Fold Change. The sign is relative to the first group identified in the comparison. lfcSE^3^ = log2 Fold change standard error. P value^4^ = Wald statistic value. Padj^5^ = Benjamini–Hochberg adjusted p value. Piglet^6^ = average relative abundance of the taxon in that row for piglets included in the trial; Sow^7^ = average relative abundance of the taxon in that row for sows included in the trial.

**Supplementary Table2**. Different viral orders between sows and piglets at weaning

| **Order** | **baseMean^1^** | **log2FoldChange^2^** | **lfcSE^3^** | **pvalue^4^** | **padj^5^** | **Piglet^6^** | **Sow^7^** |
| --- | --- | --- | --- | --- | --- | --- | --- |
| Petitvirales | 61.09 | 1.92 | 0.12 | <0.01 | <0.01 | 29.93 | 82.34 |
| Caudovirales | 36.01 | -1.98 | 0.15 | <0.01 | <0.01 | 65.88 | 12.87 |
| Cirlivirales | 1.32 | 0.64 | 0.56 | 0.25 | 0.64 | 1.19 | 1.44 |
| Cremevirales | 1.24 | 0.81 | 0.64 | 0.21 | 0.64 | 0.98 | 1.46 |
| Tubulavirales | 1.35 | 0.51 | 0.57 | 0.37 | 0.74 | 1.23 | 1.39 |
| Piccovirales | 0.20 | 0.79 | 1.34 | 0.56 | 0.93 | 0.12 | 0.23 |
| Geplafuvirales | 0.05 | 0.07 | 2.97 | 0.98 | 0.98 | 0.07 | 0.13 |
| Imitervirales | 0.11 | -0.29 | 2.19 | 0.89 | 0.98 | 0.28 | 0.00 |
| Pimascovirales | 0.02 | 0.25 | 2.97 | 0.93 | 0.98 | 0.12 | 0.02 |
| Rowavirales | 0.09 | -0.11 | 2.97 | 0.97 | 0.98 | 0.17 | 0.00 |

baseMean^1^ = mean of normalized taxa counts averaged over all samples from both conditions. log2FoldChange^2^ = log2 Fold Change. The sign is relative to the first group identified in the comparison. lfcSE^3^ = log2 Fold change standard error. P value^4^ = Wald statistic value. Padj^5^ = Benjamini–Hochberg adjusted p value. Piglet^6^ = average relative abundance of the viral order for piglets included in the trial; Sow^7^ = average relative abundance of the viral order in that for sows included in the trial.
